# Supplementary figures and images for: Recessive pathogenic variants in MCAT cause combined oxidative phosphorylation deficiency
Source: eLife. 2023 Mar 7;12:e68047. doi: 10.7554/eLife.68047 (PMC9991045; doi:10.7554/eLife.68047)

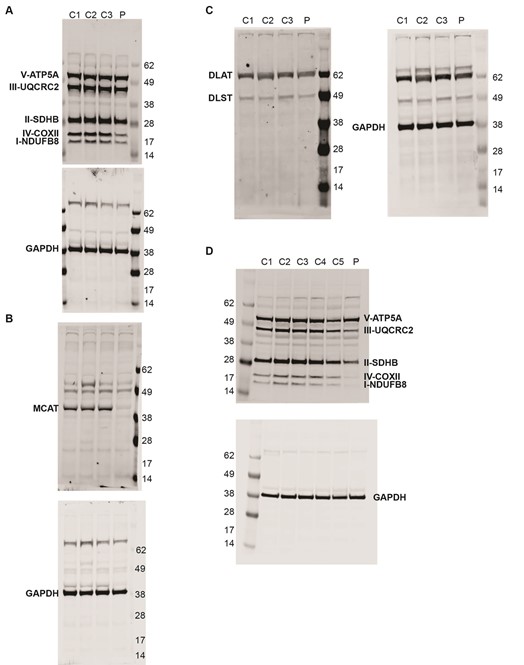

Supplement: Figure 2—source data 1. [file elife-68047-fig2-data1.zip › Figure 2- source data 1.jpeg]

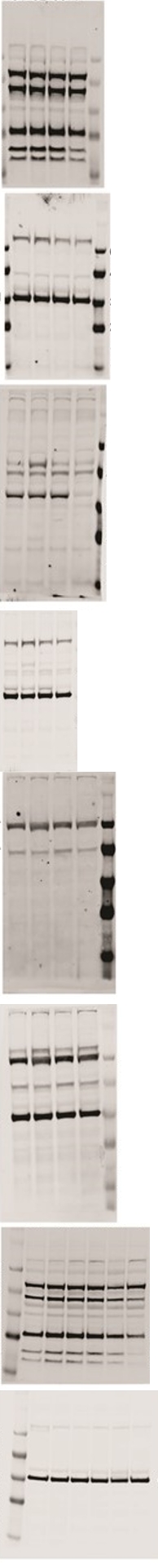

Supplement: Figure 2—source data 2. [file elife-68047-fig2-data2.zip › Figure 2- source data 2.jpg]

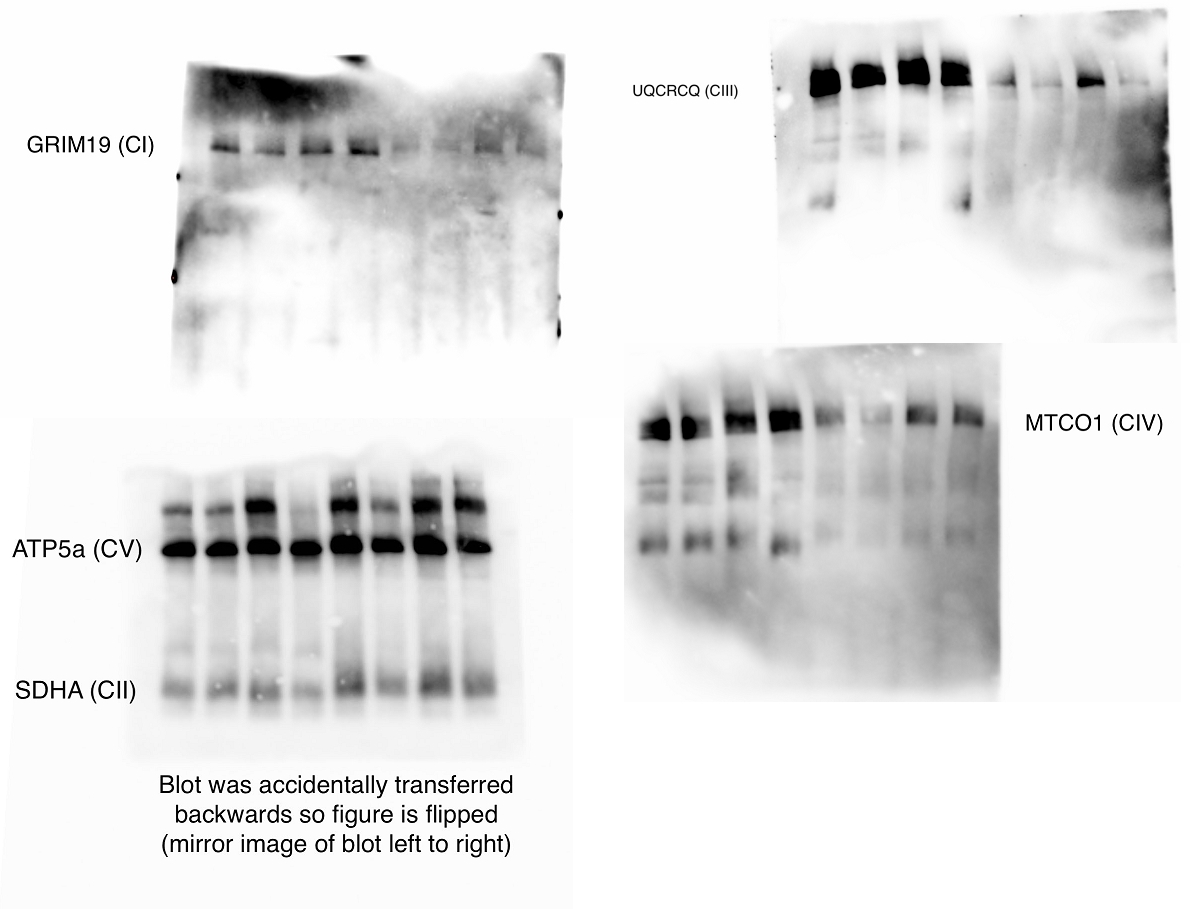

Supplement: Figure 3—source data 1. [file elife-68047-fig3-data1.zip › Figure 3-source data 1.jpg]

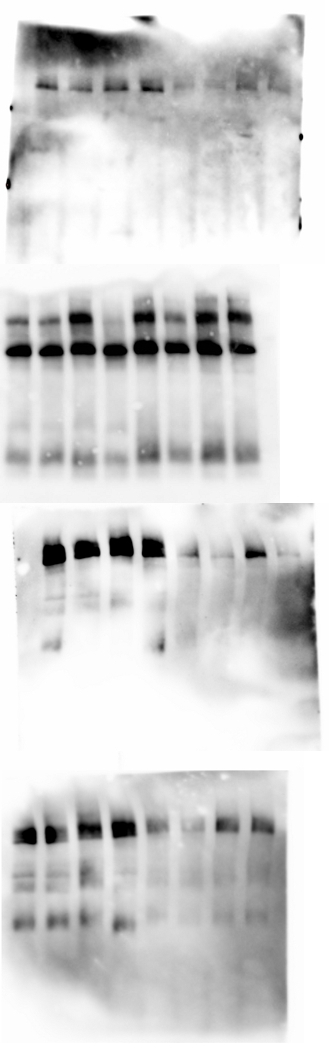

Supplement: Figure 3—source data 2. [file elife-68047-fig3-data2.zip › Figure 3-source data 2.jpg]

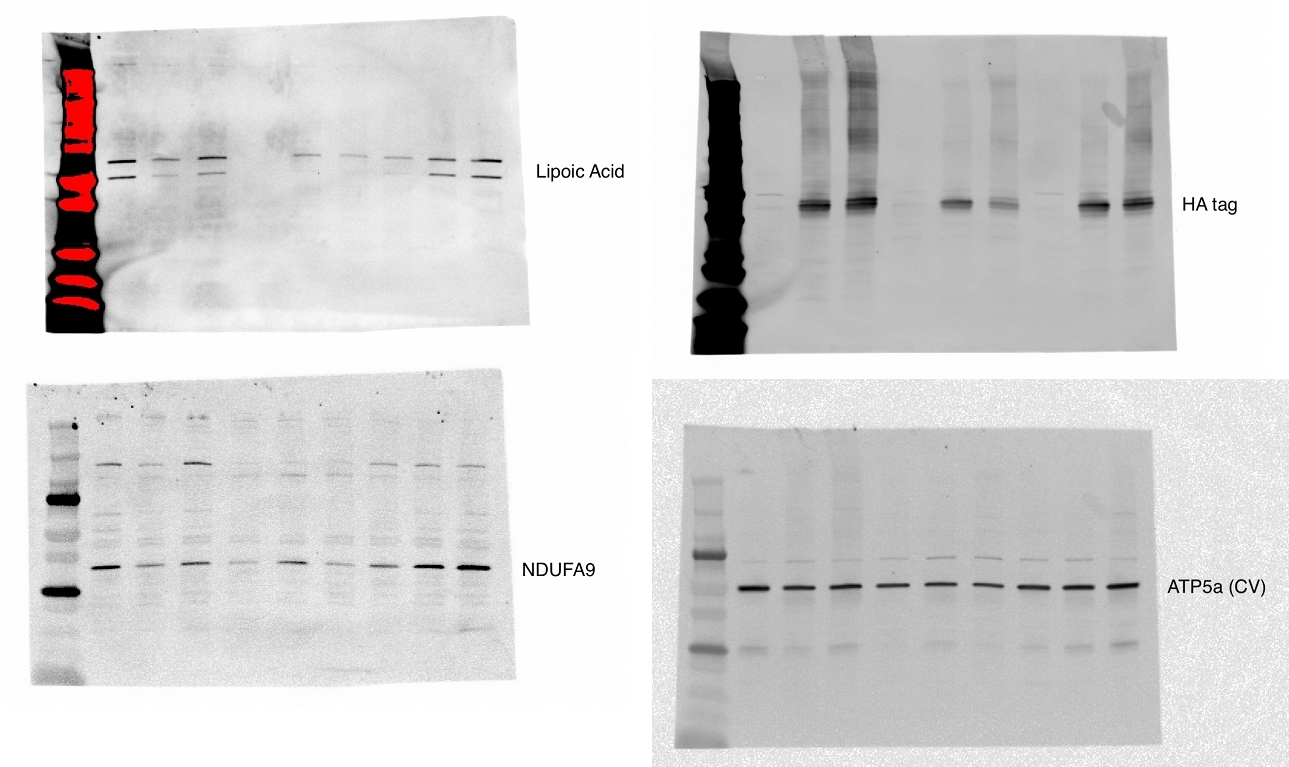

Supplement: Figure 4—source data 1. [file elife-68047-fig4-data1.zip › Figure 4- source data 1.jpg]

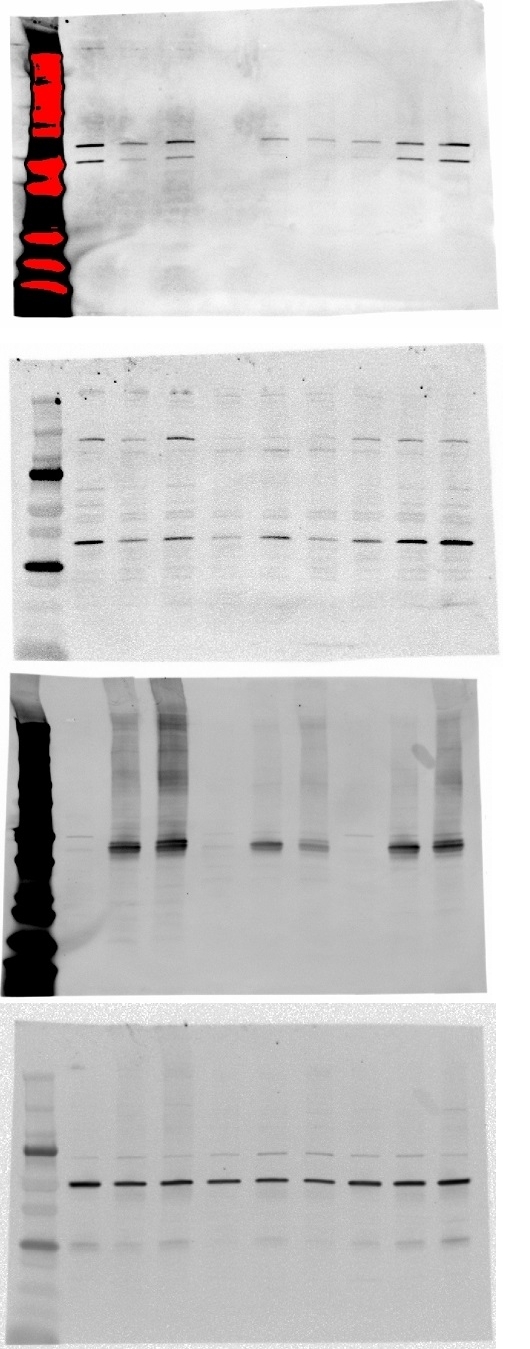

Supplement: Figure 4—source data 2. [file elife-68047-fig4-data2.zip › Figure 4- source data 2.jpg]
